# Analysis of characteristics of and risk factors for otological symptoms after COVID-19 infection

**DOI:** 10.1371/journal.pone.0297100

**Published:** 2024-02-01

**Authors:** Qiang Wang, Hailing Gu, Jianjun Ren, Yu Zhao, Zhaoli Meng

**Affiliations:** 1 Department of Otorhinolaryngology-Head & Neck Surgery, West China Hospital, Sichuan University, Chengdu, China; 2 Department of Audiology and Speech Language Pathology, West China Hospital, Sichuan University, Chengdu, China; University Putra Malaysia, MALAYSIA

## Abstract

The purpose of this study was to explore the characteristics of and risk factors for otological symptoms after contracting COVID-19. We invited 468 participants who had been infected with COVID-19 to participate in a survey. 310 (66.2%) were women and 158 (33.8%) were men. The mean age is 38.73 (12.21) years. The questionnaire included their basic information, symptoms and symptom duration after SARS-CoV-2 infection, number of vaccine doses received, and details regarding otological symptoms. In total, 106/468 (22.6%) participants experienced tinnitus, 66/468 (14.1%) hearing loss, 103/468 (22.0%) aural fullness, and 71/468 (15.2%) dizziness. Women were more prone to experience tinnitus (*P* = 0.022) and dizziness (*P* = 0.001) than men. The group with hearing loss were older (*P* = 0.025), and their initial COVID-19 symptoms lasted longer (*P* = 0.028) than those of patients without. Patients with aural fullness were more likely to experience fatigue than patients without (*P* = 0.002). Patients experiencing dizziness were more likely to experience pharyngalgia (*P* = 0.040) and fatigue (*P* = 0.005) than those without. The number of vaccine doses was positively associated with the resolution of otological symptoms (*P* = 0.035). Multiple logistic regression analysis revealed that sex was an independent risk factor for tinnitus (odds ratio [OR], 1.802; 95% confidence interval [CI], 1.099–2.953; *P* = 0.020), the duration of initial COVID-19 symptoms for hearing loss (OR, 1.055; 95% CI, 1.008–1.105; *P* = 0.023), and sex for dizziness (OR, 2.870; 95% CI, 1.489–5.535; *P* = 0.002). Sex, age, COVID-19-related fatigue, and the duration of initial COVID-19 symptoms may affect the occurrence of otological symptoms, and vaccines may aid their resolution.

## Introduction

Over 767 million confirmed cases and over 6.9 million deaths due to severe acute respiratory syndrome coronavirus 2 (SARS-CoV-2) infection have been reported globally as of 4 June 2023 [1], affecting people of all ages [2]. In December 2022, with the Chinese government’s new epidemic prevention policy, the number of SARS-CoV-2 infections in China increased significantly [3]. Most people infected with the virus experience mild to moderate respiratory symptoms, including rhinorrhea, pharyngalgia, headache, fatigue, and sneezing [4]. As the infection rates peaked, many patients were referred to our ear, nose, and throat department with these common symptoms.

Various otological and vestibular symptoms may occur within one month of SARS-CoV-2 infection [5]. Sudden sensorineural hearing loss (SSNHL) and tinnitus are prevalent among patients who have or previously had coronavirus disease 2019 (COVID-19) [6]. COVID-19 damages the inner ear, including the organ of Corti, stria vascularis, and spiral ganglion [7]; however, its spontaneous course remains unclear [8]. Chern et al. reported on an 18-year-old patient with a SARS-CoV-2 infection who presented with bilateral SSNHL, aural fullness, and dizziness [9]. Dizziness is a common symptom of COVID-19 [10]. Although autonomic dysfunction has frequently been observed in patients during and after SARS-CoV-2 infection [11, 12], the effect of SARS-CoV-2 on the vestibular system is unclear [13]. Most studies on these symptoms are case reports [14, 15], and we are aware of the few studies in which the epidemiology and morbidity of otological symptoms after SARS-CoV-2 infection have been described. Hence, the risk factors for the development of these otological symptoms and their pathogenesis and prevalence remain unclear.

Therefore, with the present study, we aimed to explore the characteristics and risk factors of otological symptoms after SARS-CoV-2 infection, specifically the prevalence, severity, and duration of symptoms and their relationship with sex, age, and vaccine dose, to lay the groundwork for patient counselling and prognostication.

## Materials and methods

### Ethics

Ethics approval was obtained from the West China Hospital of Sichuan University Biomedical Research Ethics Committee (no. 2023(137)). We included only those individuals who were able to cooperate with the survey and volunteered and provided informed consent for participation in this study. This study was conducted in the community. The researcher explained to the individuals that the research questionnaire required completion, was ethicallyapproved, and that their responses would be kept confidential. Once an individual verbally agreed to participate in the study, they were included in the study. The study did not involve minors.

### Study participants

We invited individuals over 18 years of age who had been infected with COVID-19 to participate in this study from 20 January to 28 February 2023. According to the “Diagnosis and Treatment Plan for the Novel Coronavirus Disease” version 10 [16], participants were considered to have COVID-19 if they had a positive result for a SARS-CoV-2 nucleic acid test [17] or if they had a positive result for a rapid antigen self-test [18] and had self-reported typical symptoms. Participants who do not have access to nucleic acid testing or antigen testing will be judged to be infected with COVID-19 based on self-reported symptoms and the timing of symptom onset [19]. The exclusion criteriawere having had another viral infection or disease in the previous three months or being unable to cooperate with the investigation owning to the presence of other diseases. Patients with otological symptoms before before they had COVID-19 were also excluded. To avoid selection bias in the results, the subjects were not collected in the otolaryngology department. Rather, they were randomly recruited from the three communities in Chengdu, China. This research was conducted based on an online survey. We posted instructions and two-dimensional codes for the survey in each community. Participants voluntarily scanned the codes to take the survey. To further avoid selection bias, the only information we provided participants before completing the questionnaire was that this was a survey about COVID-19, which was open to all patients with COVID-19. In accordance with ethical requirements, the full purpose of the survey was displayed at the end of the questionnaire when the participant had finished completing it, and the participant could decide whether to submit it. The procedures followed were in accordance with the ethical standards of the institutional committee on human experimentation and with the Helsinki Declaration of 1975, as revised in 2000.

### Questionnaires

All participants were asked to complete a questionnaire approximately one month after being infected with SARS-CoV-2. Participants could complete the questionnaire via their mobile phone or other device with which they could access the Internet. The content of the questionnaire was prepared in Mandarin by otolaryngology, head, and neck surgeons of West China Hospital, according to the purpose of the study.

The questionnaire comprised the following items: age; sex; method of COVID-19 confirmation; symptoms experienced while having COVID-19 (fever, nasal obstruction, pharyngalgia, smell and taste disorders, fatigue, muscular pain, and cough); duration of the above symptoms; number of COVID-19 vaccine doses received; otological symptoms experienced while having COVID-19 (tinnitus, hearing loss, aural fullness, and dizziness); time from contracting COVID-19 to the appearance of otological symptoms; whether the otological symptoms had disappeared; and duration of otological symptoms. Participants could choose from a list of options under each question or provide text, as appropriate. Questionnaires with missing data were excluded from the analyses. Symptoms relied on patient self-report. We considered participants to be experiencing dizziness when patients presented with vertigo, disequilibrium, presyncope, or lightheadedness [20]. We considered participants to have experienced hearing loss when they reported changes in their hearing levels following infection with COVID-19.

### Statistical analysis

All continuous variables are presented as means and standard deviations (SDs), and categorical data are presented as frequencies (percentages). Different otological symptoms were grouped for separate analyses. The chi-square test was used to compare categorical variables among groups, and Student’s t-test was used to compare continuous variables between groups. For each type of otological symptom, we included sex, age, vaccination doses, and duration of initial symptoms of COVID-19 as variables in a binary logistic regression model to determine their relationship with the symptom. We specifically included factors that may affect the appearance of symptoms to explore the characteristics of otological symptoms while a patient has COVID-19. We used the Mann–Whitney test to compare the differences in vaccination between patients in whom otological symptoms disappeared and those in whom such symptoms did not disappear. The number of positive answers for each otological symptom had to be more than 10 times the number of independent variables [21]. As we included four risk factors in the logistic regression analysis, the number of people positive for each otological symptom had to be no less than 40. Data analyses were performed using SPSS Statistics for Windows, Version 21.0, (IBM, Armonk, NY, USA) and GraphPad Prism software, Version 8.4.3 (GraphPad Software, San Diego, CA, USA). All statistical tests were two-sided, and *P* values < 0.05 were regarded significant.

## Results

**Fig 1:**
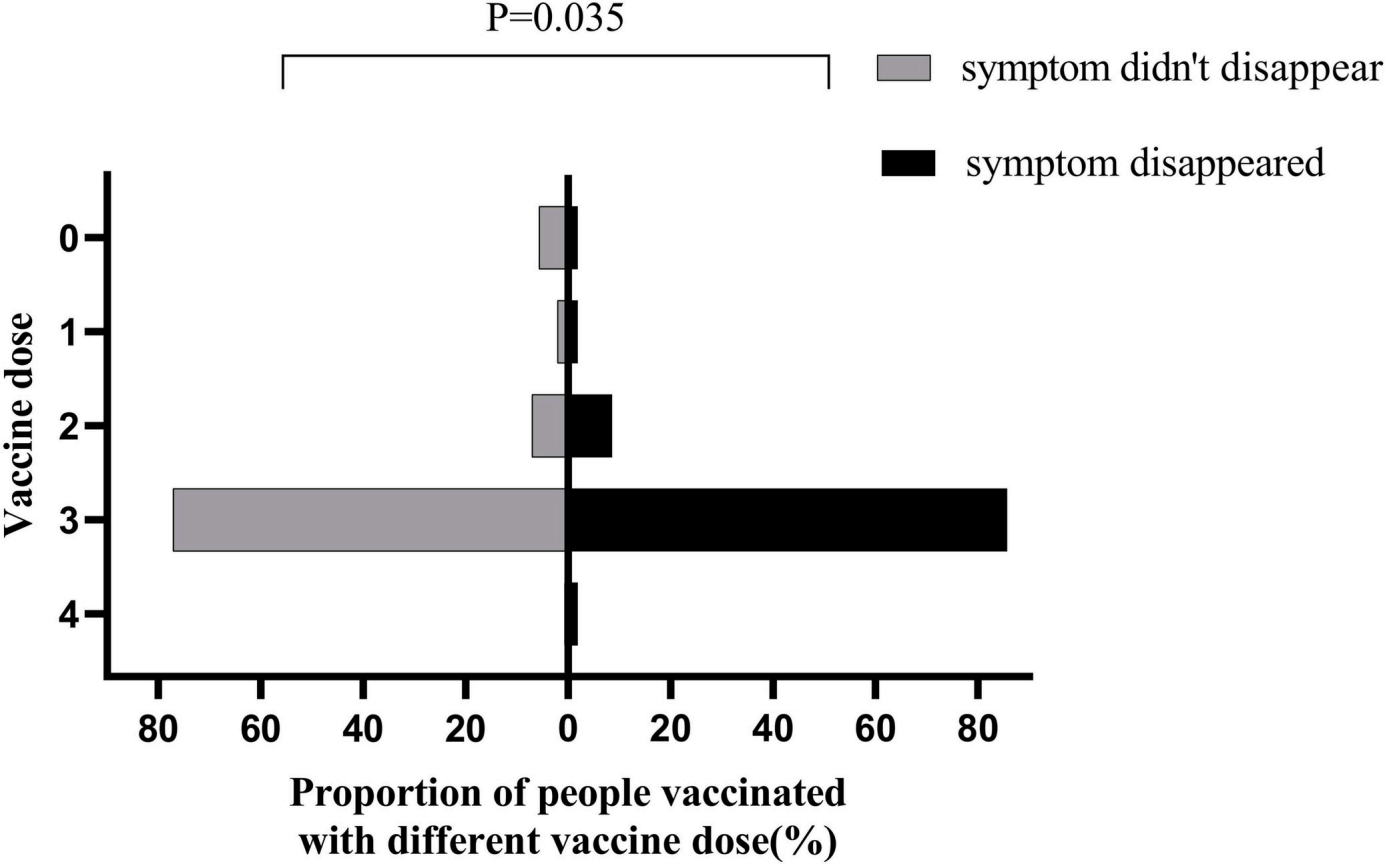
Differences in numbers vaccine doses between patients for whom otological symptoms disappeared and those for whom symptoms persisted.

A total of 472 people participated in this study, of whom 4 were excluded owing to missing data. The validity of the questionnaire was 99.15%. The baseline characteristics of the 468 included participants are summarized in Table 1. Among them, 310 (66.2%) were women and 158 (33.8%) were men. The mean age is 38.73 (12.21) years. Among the 468 participants, 19 (4.1%) were not vaccinated, 7 (1.5%) received only one dose of vaccine, 58 (12.4%) received two doses, 378 (80.8%) received three doses, and 6 (1.3%) received four doses. Of all the participants, 245 (52.4%) developed otological symptoms after a mean of 5.45 (SD = 5.09) days of infection with SARS-CoV-2. Among the 468 participants, 106 (22.6%) experienced tinnitus, 66 (14.1%) hearing loss, 103 (22.0%) aural fullness, and 71 (15.2%) dizziness. We compared sex, age, vaccination doses, typical COVID-19 symptoms (fever, nasal congestion, pharyngalgia, smell and taste disorders, fatigue, and chronic pain), and duration of initial COVID-19 symptoms between groups of patients with different otological symptoms (Tables 2–5). Women were more prone to experience tinnitus than men (*P* = 0.022), whereas the other factors did not differ between patients with and those without tinnitus. The group with hearing loss were older (*P* = 0.025) and their initial COVID-19 symptoms lasted longer (*P* = 0.028) than those of patients without hearing loss. These groups did not differ based on any other otological symptom. Patients with aural fullness were more likely to experience fatigue than those without (*P* = 0.002); these groups did not differ in terms of other characteristics. The group experiencing dizziness had a higher prevalence of women (*P* = 0.001), whereas patients experiencing dizziness were more likely to also experience pharyngalgia (*P* = 0.040) and fatigue (*P* = 0.005) than patients without dizziness. Interestingly, the proportion of patients with dizziness who experienced taste and smell disorders was lower than that of patients without dizziness (*P* = 0.046). We analyzed the differences in sex, age, vaccine dose, and duration of initial COVID-19 symptoms between patients for whom otological symptoms resolved and those for whom otological symptoms persisted (Table 6). We discovered that the number of vaccine doses was positively associated with the resolution of otological symptoms (*P* = 0.035). None of the other factors were related to the resolution of otological symptoms. The distributions of vaccine doses in both groups is displayed in Fig 1.

**Table 1:** Baseline characteristics of participants (N = 468).

Characteristics		Overall
Sex (%)	F	310 (66.24)
	M	158 (33.76)
Age (mean (SD))		38.73(12.21)
Diagnostic criteria (%)	Positive SARS-cov-2 nucleic acid	110 (23.50)
	Rapid antigen self-test	209 (44.66)
	Self-reported	148 (31.62)
Vaccine dose (%)		
	0	19 (4.06)
	1	7 (1.50)
	2	58 (12.39)
	3	378 (80.77)
	4	6 (1.28)
Otological Symptoms		
	Tinnitus (%)	106 (22.65)
	Hearing Loss (%)	66 (14.10)
	Aural Fullness (%)	103 (22.01)
	Dizziness (%)	71 (15.17)
	Without (%)	223 (47.65)

**Table 2:** Clinical characteristics of patients, stratified according to tinnitus (N = 468).

Characteristics	With Tinnitus	Without Tinnitus	*P* Value
	N = 106	N = 362	
Sex (%)			**0.022**
F	80(75.47)	230(63.54)	
M	26(24.53)	132(36.46)	
Age (years, mean (SD))	39.61(11.78)	38.47(12.22)	0.385
Vaccine Dose (%)			0.750
0	5(4.72)	14(3.87)	
1	1(0.94))	6(1.66)	
2	13(12.26)	45(12.43)	
3	87(82.08)	291(80.39)	
4	0(0.00)	6(1.66)	
Fever(%)			0.845
With	91(85.85)	308(85.08)	
Without	15(14.15)	54(14.92)	
Nasal obstruction(%)			0.699
With	75(70.75)	249(68.78)	
Without	31(29.25)	113(31.22)	
Pharyngalgia(%)			0.227
With	28(26.42)	244(67.40)	
Without	78(73.58)	118(32.60)	
Smell and taste disorders(%)			0.361
With	50(47.17)	189(52.21)	
Without	56(52.83)	173(47.79)	
Fatigue(%)			0.334
With	88(83.02)	285(78.73)	
Without	18(16.98)	77(21.27)	
Muscular pain(%)			0.854
With	71(66.98)	239(66.02)	
Without	35(33.02)	123(33.98)	
Duration of initial COVID-19 symptoms(days, mean (SD))	9.55(5.33)	9.13(5.17)	0.477

**Table 3:** Clinical characteristics of patients, stratified according to hearing loss (N = 468).

Characteristics	With Hearing Loss	Without Hearing Loss	*P* Value
	N = 66	N = 402	
Sex (%)			0.138
F	49(74.24)	261(64.93)	
M	17(25.76)	141(35.07)	
Age (years, mean (SD))	41.83(15.27)	38.22(11.48)	**0.025**
Vaccine Dose (%)			0.236
0	5(7.58)	14(3.48)	
1	3(4.55)	4(1.00)	
2	7(10.61)	51(12.69)	
3	50(75.76)	328(81.59)	
4	1(1.52)	5(1.24)	
Fever(%)			0.395
With	54(81.82)	345(85.82)	
Without	12(18.18)	57(14.18)	
Nasal obstruction(%)			0.507
With	48(72.73)	276(68.66)	
Without	18(27.27)	126(31.34)	
Pharyngalgia(%)			0.490
With	43(65.15)	279(69.40)	
Without	23(34.85)	123(30.60)	
Smell and taste disorders(%)			0.053
With	25(37.88)	198(49.25)	
Without	41(62.12)	204(50.75)	
Fatigue(%)			0.147
With	57(86.36)	316(78.61)	
Without	9(13.64)	86(21.39)	
Muscular pain(%)			0.108
With	38(57.58)	272(67.66)	
Without	28(42.42)	130(32.34)	
Duration of initial COVID-19 symptoms(days, mean (SD))	10.71(5.93)	8.98(5.04)	**0.028**

**Table 4:** Clinical characteristics of patients, stratified according to aural fullness (N = 468).

Characteristics	With aural fullness	Without aural fullness	*P* Value
	(N = 103)	(N = 365)	
Sex (%)			0.067
F	76(73.79)	234(64.11)	
M	27(26.21)	131(35.89)	
Age (years, mean (SD))	39.38 (11.33)	38.54 (12.36)	0.518
Vaccine Dose (%)			0.951
0	5(4.85)	14(3.84)	
1	1(0.97)	6(1.64)	
2	11(10.68)	47(12.88)	
3	86(83.50)	292(80.00)	
4	0(0.00)	6(1.64)	
Fever(%)			0.052
With	94(91.26)	305(83.56)	
Without	9(8.74)	60(16.44)	
Nasal obstruction(%)			0.106
With	78(75.73)	119(32.60)	
Without	25(24.27)	246(67.40)	
Pharyngalgia(%)			0.835
With	70(67.96)	252(69.04)	
Without	33(32.04)	113(30.96)	
Smell and taste disorders(%)			
With	63(61.17)	176(48.22)	0.02
Without	40(38.83)	189(51.78)	
Fatigue(%)			**0.002**
With	93(90.29)	85(23.29)	
Without	10(9.71)	280(76.71)	
Muscular pain(%)			0.410
With	72(69.9)	238(65.21)	
Without	31(30.1)	127(34.79)	
Duration of initial COVID-19 symptoms(days, mean (SD))	9.63 (5.35)	9.11 (5.16)	0.380

**Table 5:** Clinical characteristics of patients, stratified according to dizziness (N = 468).

Characteristics	With Dizziness	Without Dizziness	*P* Value
	N = 71	N = 397	
Sex (%)			**0.001**
F	59(83.10)	261(65.74)	
M	12(16.90)	141(35.52)	
Age (years, mean (SD))	38.25(10.60)	38.81(12.37)	0.694
Vaccine Dose (%)			0.479
0	3(4.23)	16(4.03)	
1	2(2.82)	5(1.26)	
2	10(14.08)	48(12.09)	
3	55(77.46)	323(81.36)	
4	1(1.41)	5(1.26)	
Fever(%)			0.847
With	60(84.51)	339(85.39)	
Without	11(15.49)	58(14.61)	
Nasal obstruction(%)			0.056
With	56(78.87)	268(67.51)	
Without	15(21.13)	129(32.49)	
Pharyngalgia(%)			**0.005**
With	59(83.10)	263(66.25)	
Without	12(16.90)	134(33.75)	
Smell and taste disorders(%)			**0.046**
With	27(38.03)	195(49.12)	
Without	44(61.97)	202(50.88)	
Fatigue(%)			**0.040**
With	63(88.73)	310(78.09)	
Without	8(11.27)	87(21.91)	
Muscular pain(%)			0.104
With	53(74.65)	257(64.74)	
Without	18(25.35)	140(35.26)	
Duration of initial COVID-19 symptoms(days, mean (SD))	10.14(5.82)	9.06(5.06)	0.148

**Table 6:** Clinical characteristics of patients, stratified according to symptoms disappeared (N = 245).

Characteristics	Symptoms disappeared	Symptoms did not disappear	*P* Value
	N = 105	N = 140	
Sex (%)			0.607
F	82(78.10)	104(74.29)	
M	24(21.90)	36(25.71)	
Age (years, mean (SD))	37.93 (11.03)	40.84(13.68)	0.068
Vaccine Dose (%)			**0.035**
0	2(1.90)	8(5.71)	
1	2(1.90)	3(2.14)	
2	9(8.57)	10(7.14)	
3	90(85.71)	108(77.14)	
4	2(1.90)	1(0.71)	
Duration of initial COVID-19 symptoms (days, mean (SD))	9.22(4.99)	9.5(5.2)	0.669

**Fig 2:**
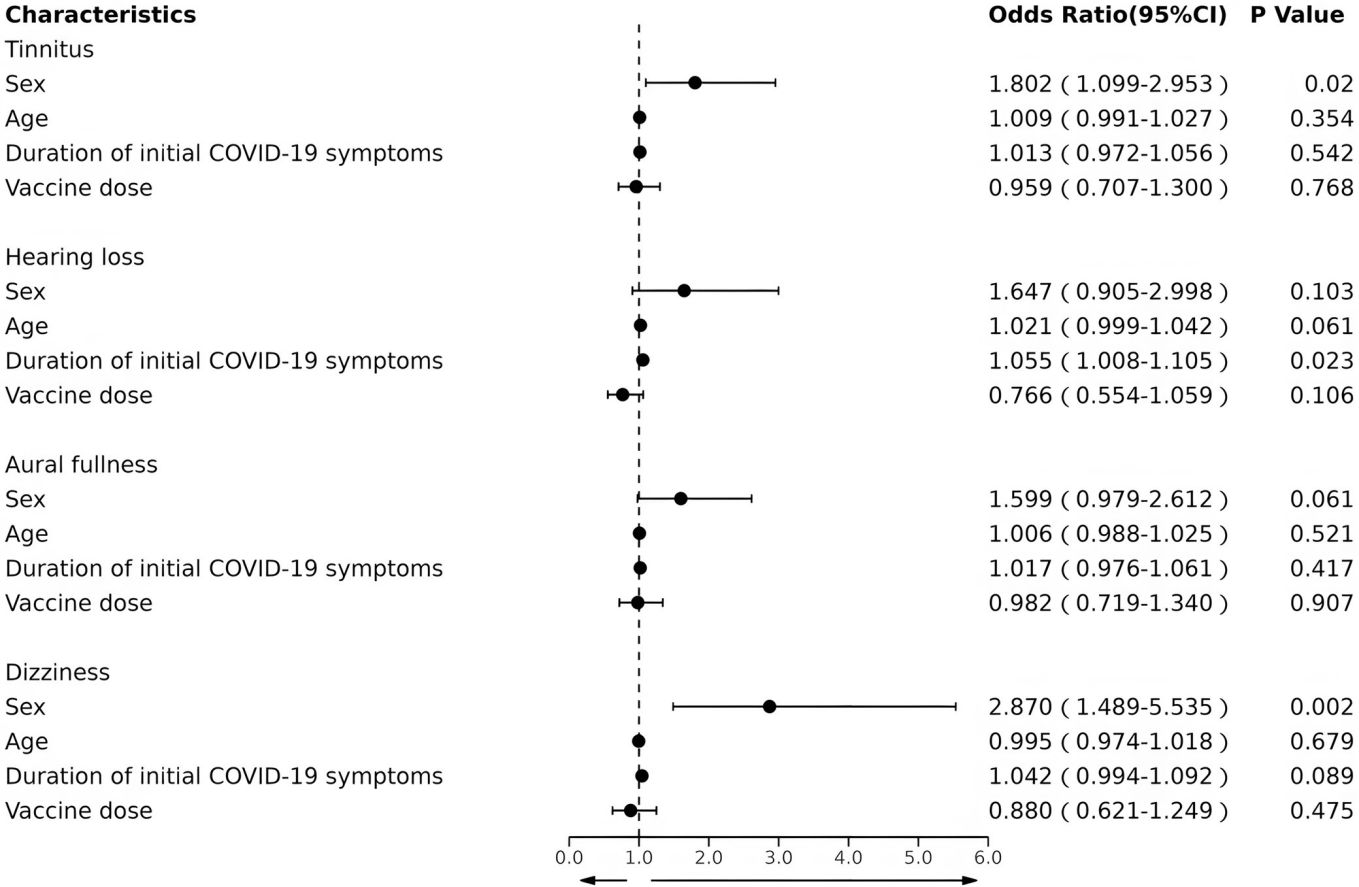
Multiple logistic regression for the occurrence of different otological symptoms.

The multiple logistic regression analysis of the different otological symptoms is summarized in Fig 2. Sex was an independent risk factor for tinnitus (odds ratio, 1.802; 95% confidence interval, 1.099–2.953; *P* = 0.020). The probability of tinnitus was 1.802 times higher in women than in men. The duration of initial COVID-19 symptoms was an independent risk factor for hearing loss (odds ratio, 1.055; 95% confidence interval, 1.008–1.105; *P* = 0.023). This means that, for every extra day of initial COVID-19 symptoms, the probability of hearing loss increased by 5.5%. None of the factors included in the model were associated with aural fullness (all *P*>0.05). Finally, sex was an independent risk factor for dizziness (odds ratio, 2.870; 95% confidence interval, 1.489–5.535; *P* = 0.002). The probability of experiencing dizziness was 2.87 times more likely for women than for men.

## Discussion

COVID-19 has had a huge impact on human health worldwide. As the virus continues to evolve, the site of viral invasion is gradually shifting from the lungs to the upper respiratory tract [21]. Therefore, increasing attention has been paid to otorhinolaryngological symptoms after infection with SARS-CoV-2. Previous studies have revealed that COVID-19, especially prolonged infections, may cause tinnitus, hearing loss, and dizziness [22–24]. In this study, we explored the characteristics of and risk factors for otological symptoms following SARS-CoV-2 infection. We observed that sex, age, the occurrence of typical symptoms of COVID-19, the duration of initial symptoms of COVID-19, and the number of vaccine doses were related to the occurrence and disappearance of otological symptoms after SARS-CoV-2 infection. This is of great importance for the prevention and treatment of COVID-19-related otological symptoms.

Our study revealed that women with COVID-19 may be more prone to tinnitus and dizziness than men, which may be owing to differences in vestibular function between women and men [25]. An increasing number of studies are demonstrating that the effects of COVID-19 differ according to sex and age. For instance, COVID-19 may adversely affect the male reproductive system [26]. The probability of otolaryngological symptoms in women after SARS-CoV-2 infection may also be greater than that in men [27]. In our study, older patients were more prone to hearing loss than younger patients. Certain studies have suggested that older people have more severe symptoms after SARS-CoV-2 infection than younger people [28]. This may be related to the weakening of the immune system with age [29]. Therefore, we suggest that women and the elderly should especially pay attention to their health after having COVID-19. Medical interventions should be planned according to sex and age for patients with COVID-19.

Our study also revealed that COVID-19-related fatigue may be related to the occurrence of certain otological symptoms. Fatigue seems to be one of the long-term effects of COVID-19 on the human body [30]. Inflammatory markers of patients who experience COVID-19-related fatigue were significantly increased in certain studies [31]. Aural fullness and dizziness may be manifestations of otitis media and nervous tissue inflammation following SARS-CoV-2 infection [32, 33]. This may explain our result that people with fatigue after infection with SARS-CoV-2 are more likely to suffer from aural fullness and dizziness. We also discovered that the a longer duration of the initial symptoms of COVID-19 increased the risk of hearing loss, which may be related to the severity of the infection. Prolonged COVID-19 increases the probability that the virus will attack the auditory system. However, as this was a retrospective study, the results of symptom duration might have been influenced by patient recall bias. Although this result should be interpreted with caution, timely symptomatic treatment and prevention of symptoms are likely necessary.

Our study complements the observed positive effect of COVID-19 vaccines on otological symptoms after contracting COVID-19. The number of vaccine doses a patient received was positively related to the resolution of otological symptoms in our study. Vaccines can rapidly eliminate viruses by activating the human immune system to produce antibodies [34]. Vaccines are known to reduce SARS-CoV-2 infections and especially to prevent the symptoms of COVID-19 [35]. However, the protective effect of vaccines on the body is time-sensitive and reportedly greatly weakens after 6 months [36]. Therefore, we suggest that people should be vaccinated with the COVID-19 vaccine within this period for symptom prevention and relief.

In this study, we discovered the characteristics of and certain risk factors for otological symptoms after contracting COVID-19, which may help us to better cope with the impact of COVID-19 on humans. According to the results of the study, women and older adults should be alerted to the potential development of otological symptoms after contracting COVID-19. Medical support should be promptly provided when symptoms develop. In addition, all people should consider being vaccinated against COVID-19. Our evidence suggests that the COVID-19 vaccine has a positive effect on the disappearance of otological symptoms.

However, our study has several limitations. First, as our study relied on patient questionnaire results, it was subject to recall bias. This may affect the accuracy of the results, especially in relation to symptom duration. Whether the patient can understand the items of the questionnaire will also affect our results. Moreover, as our study relied on patient self-reporting, we did not verify the rigor of their medical examinations, and symptoms might have been underreported. We can’t know the type or degree of hearing loss due to lack of hearing tests. Other confounders of participants’ post-infection on otologic symptoms such as previous occupational exposure to noise, hypertension, diabetes mellitus, previous or recent administration of ototoxic antibiotics were not considered in this study. At the same time, we only considered confounding factors before the participant contracted COVID-19 and did not investigate factors that might have affected otological symptoms after they contracted the disease, which biased our results to some extent. Furthermore, owing to the lack of economic and medical resources, some of the included patients were not diagnosed with COVID-19 via nucleic acid testing or antigen testing, and only their self-reported symptoms and environmental factors were used to judge whether they had COVID-19. In addition, the prevalence rate of COVID-19 was quite high and might have been overestimated. Owing to our survey taking the form of a questionnaire, people with symptoms might have been more interested in completing the questionnaire. What’s more, as the survey was conducted online, we can’t konw the response rate to the questionnaire. The present study was also limited by the sample size, so studies based on larger populations are necessary.

## Conclusion

In conclusion, the analysis of people who had been infected with COVID-19 revealed the presence of tinnitus, hearing loss, aural fullness, dizziness, and other otological symptoms. Our results lead us to suggest that women, the elderly, and those with prolonged initial symptoms of COVID-19 should pay special attention to the emergence of otological symptoms after SARS-CoV-2 infection and seek timely medical support. Finally, timely and comprehensive vaccination may aid in the resolution of otological symptoms.
